# Safety evaluation of *Lactococcus lactis* IDCC 2301 isolated from homemade cheese

**DOI:** 10.1002/fsn3.2648

**Published:** 2021-11-17

**Authors:** Taeok Kim, Shakti Chandra Mondal, Chae‐Rim Jeong, So‐Rim Kim, O‐Hyun Ban, Young Hoon Jung, Jungwoo Yang, Soo‐Jung Kim

**Affiliations:** ^1^ Department of Integrative Food, Bioscience and Biotechnology Chonnam National University Gwangju Republic of Korea; ^2^ Ildong Bioscience Gyeonggi‐do Republic of Korea; ^3^ School of Food Science and Biotechnology, and Institute of Fermentation Biotechnology Kyungpook National University Daegu Republic of Korea

**Keywords:** homemade cheese, *Lactococcus lactis* IDCC2301, probiotics, safety evaluation

## Abstract

For applications of microorganisms as probiotics in the food industry, safety evaluation has increasingly become important to ensure the health of consumers. Although people have been using various lactic acid bacteria for different purposes, some studies have reported that certain lactic acid bacteria exhibit properties of virulence and produce toxic compounds. Thus, it is necessary to examine the characteristics associated with lactic acid bacteria that are safe for use as probiotics. This research aimed to assess the safety of *Lactococcus lactis* IDCC 2301 isolated from homemade cheese using in vitro and in vivo assays, including antibiotic resistance, hemolytic activity, toxin production, infectivity, and metabolic activity in immune‐compromised animal species. The results demonstrated that the strain was susceptible to nine antibiotics suggested by the European Food Safety Authority (EFSA). Whole‐genome analysis revealed that *L. lactis* IDCC 2301 neither has toxigenic genes nor harbors antibiotic resistance. Moreover, *L. lactis* IDCC 2301 showed neither hemolytic nor β‐glucuronidase activity. Furthermore, none of the D‐lactate and biogenic amines were produced by *L. lactis* IDCC 2301. Finally, it was demonstrated that there was no toxicity and mortality using single‐dose oral toxicity tests in rats. These results indicate that *L. lactis* IDCC 2301 can be safely used as probiotics for human consumption.

## Graphical abstract

Safety assessment of *Lactococcus lactis* IDCC 2301 isolated from homemade cheese was performed. It was proven that *L. lactis* IDCC 2301 can be safely used as probiotics for human consumption through in vitro and in vivo experiments about safety.

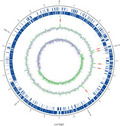

## 1 INTRODUCTION

Probiotics can be defined as “live microorganisms, which when administered in adequate amounts confer a health benefit to the host” (FAO/WHO, 2002). To date, probiotics have become commercially available as nutritional supplements in the form of various food products (Plessas et al., 2012). Because microorganisms derived from food fermentation have been considered safe and are generally referred to as Generally Recognized As Safe (GRAS) microorganisms, the majority of lactic acid bacteria (LAB) and bifidobacteria can be generally considered as safe for use (Holzapfel et al., 1998; Leroy and Vuyst, 2004; Wessels et al., 2004). Nevertheless, probiotics, including newly isolated strains, need to be characterized in terms of more safety aspects to ensure they are safe for human consumption (Huys et al., 2013). An FAO/WHO working team suggested that consequence tests, including metabolic activities, antibiotic resistance, toxin production, hemolytic activities, infectivity in immunocompromised animal models, side effects in humans, and adverse outcomes in consumers, are needed to assess the characteristics of the probiotic strains to establish safety guidelines for probiotic organisms (FAO/WHO, 2002). It is already established that the isolation history and species identification of the probiotic source should be covered in safety aspects and provide evidence of phenotypic and/or genotypic characteristics exhibiting that the culture does not gain antibiotic resistance traits, other pathogenic properties, or putative virulence factors (Salminen et al., 1998; Sanders et al., 2010; Vankerckhoven et al., 2008). Recent interests of probiotics increase due to scientific evidences concerning health benefits. Thus, the Korean market grew fast: from $140 million (USD) in 2015 to $770 million in 2019. Furthermore, interests of consumers are also escalating in terms of the safety of probiotics as a functional food. In accordance with this notion, *Lactococcus lactis* IDCC 2301 strain which is isolated from homemade cheese and commercially available is required to evaluate and assess the safety as a probiotic product.

In this study, *L. lactis* IDCC 2301 was assessed for safety measures using genotypic and phenotypic methods to fulfill the recommendation of the EFSA manual. First, in vitro tests were performed to determine the minimal inhibitory concentrations (MICs) against various antibiotics and the hemolytic and enzymatic activities of *L. lactis* IDCC 2301. Whole‐genome analysis was carried out to determine whether this strain has genes associated with toxigenic and antibiotic resistance and whether these genes can be transferred to commensal or pathogenic bacteria. Also, we determined carbohydrate patterns, biogenic amine production, and lactate formation. For in vivo test, a single‐dose oral toxicity test was performed in rats.

## 2 MATERIALS AND METHODS

### 2.1 Culture conditions of *L. lactis*

The *L. lactis* IDCC 2301 strain was isolated from homemade cheese and cultured facultative anaerobically in MRS medium (BD Difco) at 37°C. Cell growth was monitored by measuring the absorbance at 600 nm using a microplate spectrophotometer (BioTek).

### 2.2 Determination of carbohydrate fermentation patterns

The carbohydrate fermentation patterns of *L. lactis* IDCC 2301 were investigated using an API50 CHL/CHB kit (BioMérieux) containing 49 selected carbohydrate sources according to the manufactures’ manual. Cells grown in MRS medium overnight were obtained after centrifugation and suspended in 10 mL of API 50 CHL medium. The concentration of cells was adjusted to 6.0 × 10^8^ CFU/mL and then applied to cupules with different carbohydrates on an API 50 CHL test strip. The patterns of fermentation were determined for up to 48 h at 37°C. The color changes, which represent carbohydrate fermentation, were evaluated.

### 2.3 Examination of antibiotic susceptibility

The susceptibility of *L. lactis* IDCC 2301 to various antibiotics, which are commonly used for the treatment of enterococcal infections, was evaluated. In this test, nine antibiotics were used, namely ampicillin, clindamycin, chloramphenicol, erythromycin, gentamicin, kanamycin, streptomycin, tetracycline, and vancomycin. In brief, the cells were grown overnight, and then, 1% (*v*/*v*) of cells was transferred to fresh MRS broth. When the cell density reached approximately 10^6^ CFU/mL, 50 μL of culture broth and 50 μL of each antibiotic solution at various concentrations were transferred to a 96‐well plate to obtain 3.3–6.6 × 10^5^ CFU/mL and 0.125–1024 μg/mL, respectively. Then, the plates were incubated at 37°C for 20 h, and the absorbance at 600 nm was measured using a microplate spectrophotometer (BioTek). Finally, MIC was determined as the lowest concentration in which cell growth was completely inhibited.

### 2.4 Genomic analysis of virulence factors and antibiotic genes in *L. lactis*

A PacBio RSII instrument with an Illumina platform (Macrogen) was used for whole‐genome sequencing of *L. lactis* IDCC 2301. Putative virulence factors were identified using the BLASTn algorithm based on the virulence factor database (http://www.mgc.ac.cn/VFs/) setting thresholds of identity of >70%, coverage >70%, and E‐value < 1E‐5 (Chen et al., 2005). To investigate the genes of putative antibiotic resistance, the ResFinder3.2 software was used to compare the assembled sequences with the antibiotic resistance gene sequence of reference in the ResFinder database (https://cge.cbs.dtu.dk/services/ResFinder/). For this analysis, the search parameters used were sequence identity >80% and coverage >60% (Zankari et al., 2012). The target antibiotics were as follows: aminoglycoside, beta‐lactam, colistin, fosfomycin, fusidic acid, glycopeptides, macrolide, nitroimidazole, oxazolidinone, phenicol, quinolone, rifampicin, sulfonamide, tetracycline, and trimethoprim.

### 2.5 Test of hemolysis

The cells were streaked onto sheep blood agar plates (BD Difco) and cultured at 37°C overnight. The hemolytic potential of *L. lactis* IDCC 2301 was determined by measuring the clear zones around the colonies the next day. *Staphylococcus aureus* subsp. *aureus* ATCC 25923 was used as a positive control for β‐hemolysis. The strain was cultured in a brain heart infusion medium at 37°C overnight.

### 2.6 Test for enzymatic activity

To investigate the activities of 19 enzymes in *L. lactis* IDCC 2301, the API ZYM Kit (BioMérieux) was used according to the manufacturer’s protocols. Briefly, *L. lactis* IDCC 2301 cells were cultured in MRS medium at 37°C overnight. The cells were harvested using centrifugation and suspended in phosphate‐buffered saline to adjust the final cell concentration to 1.8 × 10^9^ CFU/mL. Then, the cells were cultured in MRS medium at 37°C for 4 h before applying them to the API ZYM strips. After the reaction of the cells with the ZYM‐A and ZYM‐B reagents for 5 min, the color changes, which represent enzymatic activity, were determined using the color reaction chart.

### 2.7 Test for biogenic amine production

*L. lactis* IDCC 2301 was incubated in 10 mL of MRS medium at 37°C for 24 h. After centrifugation of the culture broth at 4000 rpm at 4°C for 30 min, the supernatant was obtained and filtered using a 0.22‐μm pore size membrane. To extract the biogenic amines, 750 μL of the supernatant was mixed with the equivalent of 0.1 M HCL and filtered using a 0.45‐μm membrane. Subsequently, 1 mL of the extracted biogenic amine was incubated in a water bath at 70°C for 10 min, followed by the addition of 20 μL of 2 M NaOH, 200 μL of saturated NaHCO_3_, and 500 μL of dansyl chloride (10 mg/mL acetone) for derivatization. The derivatized biogenic amines were mixed with 200 μL of proline (100 mg/mL H_2_O) and incubated in the dark at room temperature for 15 min. The mixture was made up to 5 mL with acetonitrile (high‐performance liquid chromatography (HPLC) grade; Sigma‐Aldrich). The derivatized biogenic amines were quantified using HPLC (LC‐NETII/ADC, Jasco) with an Athena C18 column (4.6 × 250 mm, ANPEL Laboratory Tech.). The column was eluted with aqueous acetonitrile solution (67:33 of H_2_O, *v*/*v*) at a flow rate of 0.8 mL/min. UV detector (UV‐2075 plus, Jasco) at 254 nm was used to detect the biogenic amines, and quantification of the biogenic amines was performed using the calibration curves of each biogenic amine, including cadaverine, histamine, putrescine, 2‐phenethylamine, and tyramine.

### 2.8 Measurement of lactate formation

To analyze lactate production by *L. lactis* IDCC 2301, the supernatants were obtained from *L. lactis* IDCC 2301 grown in 10 mL of MRS medium at 37°C for 24 h using centrifugation at 7000 rpm at 4°C for 30 min and filtration using a 0.22‐μm pore size membrane. To quantify L‐ and D‐lactate in the filtered supernatants, an assay kit (Megazyme) was used according to the manufactures’ instructions. The absorbance at 340 nm of the reactant was measured using a microplate reader (Bio‐Rad).

### 2.9 Acute oral toxicity of *L. lactis* IDCC 2301

Acute oral toxicity (AOT) test was carried out at the Korea Testing and Research Institute (KTR; Hwasun‐gun). The AOT test was performed according to the guidelines of the organization for economic cooperation and development (OECD) for testing chemicals. In brief, female rats were randomly divided into four groups of three rats (two groups age 9 weeks; and others age 10 weeks) (OECD, 2008). Rats in the treatment group were orally administered 300 or 2000 mg of free‐dried *L. lactis* IDCC 2301 powder in 10 mL sterilized water per kg body weight. Then, the mortality, signs of body weight changes, and toxicity were monitored for 14 days. On the 14th day, the rats were euthanized by injecting 100 mL isoflurane and an autopsy was conducted to examine the organs.

## 3 RESULTS

### 3.1 Carbohydrate fermentation ability of *L. lactis* IDCC 2301

Lactic acid bacteria can grow on a variety of monosaccharides. Carbohydrates from 49 different sources were evaluated for fermentation patterns by *L. lactis* IDCC 2301, which is presented in Table 1. Based on the sources, different fermentation capabilities were observed. After incubation at 37°C for 48 h, among the 49, only 14 sources had been used as fermentation medium by the selected bacterial strain. However, ribose and monomeric carbohydrates, such as galactose, glucose, fructose, and mannose, were utilized efficiently compared with amygdaline, salicin, and amidon, whereas other sources had not been used at all.

**TABLE 1:** Carbohydrate utilization by *Lactococcus lactis* IDCC2301

No.	Substrate	Result	No.	Substrate	Result	No.	Substrate	Result	No.	Substrate	Result
1	Glycerol	−	14	L‐Sorbose	−	27	Cellobiose	+	40	D‐Turanose	−
2	Erythritol	−	15	Rhamnose	−	28	Maltose	+	41	D‐Lyxose	−
3	D‐Arabinose	−	16	Dulcitol	−	29	Lactose	−	42	D‐Tagatose	−
4	L‐Arabinose	−	17	Inositol	−	30	Melibiose	−	43	D‐Fucose	−
5	Ribose	+	18	Mannitol	−	31	Sucrose	+	44	L‐Fucose	−
6	D‐Xylose	−	19	Sorbitol	−	32	Trehalose	+	45	D‐Arabitol	−
7	L‐Xylose	−	20	α‐Methyl‐D‐mannoside	−	33	Inulin	−	46	L‐Arabitol	−
8	Adonitol	−	21	α‐Methyl‐D‐glucoside	−	34	Melezitose	−	47	Gluconate	−
9	β‐Methyl‐xylose	−	22	N‐Acetyl‐glucosamine	+	35	D‐Raffinose	−	48	2‐keto‐gluconate	−
10	Galactose	+	23	Amygdaline	+^w^	36	Amidon	+^w^	49	5‐keto‐gluconate	−
11	D‐Glucose	+	24	Arbutine	−	37	Glycogene	−			
12	D‐Fructose	+	25	Esculin	+	38	Xylitol	−			
13	D‐Mannose	+	26	Salicin	+^w^	39	Gentibiose	+			

(+), utilization; (+^W^), weak utilization; (−) non‐utilization.

### 3.2 Determination of MIC and whole‐genome analysis

**FIGURE 1:**
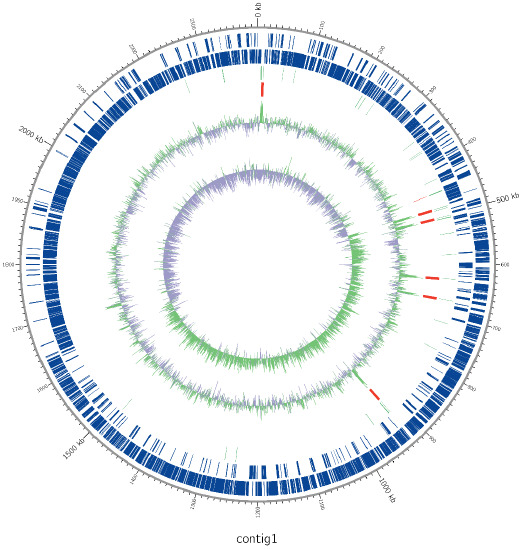
Genomic map of *Lactococcus lactis* IDCC 2301. Marked features are seen from outside to the center; CDS on the forward strand, CDS on the reverse strand, tRNA, rRNA, GC content, and GC skew

*L. lactis* IDCC 2301 was assessed to determine whether it was susceptible to the nine antibiotics that are commonly used for the treatment of enterococcal infections (EFSA & FEEDAP, 2012; Ban et al., 2020). The nine antibiotics were ampicillin, clindamycin, chloramphenicol, erythromycin, gentamicin, kanamycin, streptomycin, tetracycline, and vancomycin. We determined the following MIC values: 64 µg/mL for kanamycin, 32 µg/mL for gentamycin and streptomycin, 8 µg/mL for chloramphenicol, 4 µg/mL for vancomycin and tetracycline, 2 µg/mL for ampicillin, and 1 µg/mL for erythromycin and clindamycin, indicating that *L. lactis* IDCC 2301 was susceptible to all the antibiotics tested (Table 2). The whole‐genome sequence of *L. lactis* IDCC 2301 showed the highest similarity based on average nucleotide identity (ANI). The total length of the *L. lactis* IDCC 2301 genome was approximately 2.51 Mb with a GC content of 35.14% and 2403 functional genes (CDS) (Figure 1). In agreement with the MIC tests, the whole‐genome analysis of *L. lactis* IDCC 2301 showed that it harbored no gene or similar genes associated with antibiotic resistance in its genome (Figure 1).

**TABLE 2:** Minimum inhibitory concentration (MIC) of *Lactococcus lactis* IDCC 2301 against nine antibiotics

	AMP	CLI	CHL	ERY	GEN	KAN	STR	TET	VAN
Cutoff value^a^ (μg/mL)	2	1	8	1	32	64	32	4	4
*L. lactis* IDCC 2301	0.25/S^2^	0.125/S	4/S	<0.125/S	8/S	16/S	16/S	0.25/S	1/S

Abbreviations: AMP, ampicillin; CLI, clindamycin; CHL, chloramphenicol; ERY, erythromycin; GEN, gentamicin; KAN, kanamycin; S, Susceptible; STR, streptomycin; TET, tetracycline; VAN, vancomycin.

^a^ EFSA (European Food Safety Authority) (EFSA & FEEDAP, 2012).

### 3.3 Hemolytic property and biogenic amine production

To determine the pathogenicity of *L. lactis* IDCC 2301, an evaluation of hemolytic properties is tested. *L. lactis* IDCC 2301 was found to be negative for hemolytic activity, whereas *Staphylococcus aureus* ATCC 25923 induced β‐hemolysis on sheep blood agar as a positive control in our analysis (Table 3). Fermented foods contain a lot of biogenic compounds and they may have some biological functions, such as bioactivities that can cause a variety of adverse effects on human health (Erdag et al., 2018). In this study, we demonstrated that five types of biogenic amines, namely, cadaverine, histamine, putrescine, 2‐phenethylamine, and tyramine, were not detected in *L. lactis* IDCC 2301.

**TABLE 3:** Hemolytic activities of *L. lactis* IDCC 2301. *S. aureus* ATCC 25923 and *Lactobacillus reuteri* IDCC3701 were used as the positive and negative controls, respectively, for β‐hemolysis

	Scheme	After test
	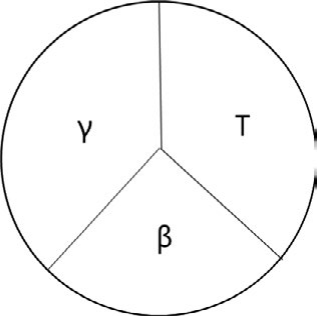	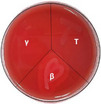
Region	Strains	Note
T sample	*L. lactis* IDCC 2301	–
β control	*S. aureus* ATCC 25923	Transparent ring by the hemolytic phenomenon
γ control	*Lb. reuteri*	No phenomenon

### 3.4 Enzymatic activity test

Since enzymatic activity of microbes is important factor for safety of probiotics, we tested various enzymatic activities including β‐glucuronidase, esterase, and lipase. In our study, *L. lactis* IDCC 2301 found to have no β‐glucuronidase activity (Table 4). However, five enzymes showed positive activities, namely, leucine arylamidase, α‐chymotrypsin, acid phosphatase, naphthol‐AS‐BI‐phosphohydrolase, and β‐glucosidase. However, most of the enzymes did not show any activity. Thus, *L. lactis* IDCC 2301 seems not to produce toxic chemicals during the fermentation.

**TABLE 4:** Enzymatic activities of *L. lactis* IDCC2301

Enzyme	Activity
Alkaline phosphate	−
Esterase	−
Esterase lipase	−
Lipase	−
Leucine arylamidase	+
Valine arylamidase	−
Cysteine arylamidase	−
Trypsin	−
α‐Chymotrypsin	+
Acid phosphatase	+
Naphthol‐AS‐BI‐phosphohydrolase	+
α‐Galactosidase	−
β‐Galactosidase	−
β‐Glucuronidase	−
α‐Glucosidase	−
β‐Glucosidase	+
N‐Acetyl‐β‐glucosaminidase	−
α‐Mannosidase	−
α‐Fucosidase	−

### 3.5 Lactate formation of *L. lactis* IDCC 2301

Lactic acid bacteria can produce lactate from pyruvate using homo‐fermentative or hetero‐fermentative methods (Drinan et al., 1976). Based on the strains and growth conditions, L‐lactate is predominantly produced; however, D‐lactate can also be produced from pyruvate (Zuniga et al., 1993). Because it is difficult for D‐lactate to be metabolized, it can accumulate in the human body, leading to D‐lactic acidosis (Petersen, 2005; Schiraldi et al., 2003). Thus, D‐lactate formation by LAB is a critical factor for safety assessment. In this study, the production of lactate by *L. lactis* IDCC 2301 was verified to be 100% of L‐lactate (Table 5).

**TABLE 5:** The ratio of l‐ to d‐lactate produced by *L. lactis* IDCC2301

Strain	l‐Lactate (mg/mL)	d‐Lactate (mg/mL)	Ratio (%)	
			L‐form	D‐form
*L. lactis* IDCC2301	18.27 ± 0.40	N.D.	100	0

Abbreviation: N.D., not detected

### 3.6 Single‐dose acute oral toxicity of *L. lactis* IDCC 2301

To evaluate the safety of *L. lactis* IDCC 2301 in vivo, single‐dose acute oral toxicity tests with four test groups were conducted for 14 days (Table 6). It was observed that a single oral dose of 0.23–1.6 × 10^12^ CFU and 0.22–1.6 × 10^12^ CFU of *L. lactis* IDCC 2301 did not cause mortality and toxicity in 9‐week‐old and 10‐week‐old rats, respectively. Moreover, there were no significant changes in the appearances, behaviors, body weight (Table 6), and feed intake of the rats. Throughout the autopsy, no gross pathological change was observed in all rats. Therefore, it was concluded that there is no evidence of any toxicity caused by *L. lactis* IDCC 2301 in the rats.

**TABLE 6:** Body weight changes in the tested rats

Dose (mg/kg B. W.^a^)	Group	Day(s) after administration				
		0	1	3	7	14
Ll^a^, 300	9‐week‐old	215.2 ± 4.7	236.4 ± 9.1	243.9 ± 5.7	256.5 ± 9.5	260.5 ± 7.0
	10‐week‐old	229.4 ± 1.0	253.2 ± 2.2	255.8 ± 3.3	264.0 ± 2.7	267.7 ± 4.8
Ll, 2000	9‐week‐old	216.5 ± 15.1	237.4 ± 20.7	242.1 ± 17.5	254.1 ± 22.4	264.8 ± 24.5
	10‐week‐old	231.6 ± 11.6	250.0 ± 11.5	257.9 ± 9.3	273.3 ± 9.3	280.4 ± 13.7

Abbreviation: BW, body weight.

^a^ Ll, *Lactococcus lactis* IDCC 2301.

## 4 DISCUSSION

Studies have shown that *L. lactis* have immense bioeconomic value because of its diverse applications in food industries. Although *L. lactis* is considered a nonpathogenic organism, care must be taken before it is used for human consumption. Recently, Karaaslan *et al*., Topcu *et al*., and Uchida *et al*. reported symptoms of *L. lactis* infection in children (Karaaslan et al., 2016; Topçu et al. 2011; Uchida et al., 2011). Hence, efficient use of lactic acid bacteria requires a complete understanding of many aspects of bacterial physiology, such as consumption of proteins and sugars for growth, lactate formation from sugars, synthesis of flavoring substances involved in cheese, and relation in different fermentations. In this study, we investigated the safety properties of *L. lactis* IDCC 2301 isolated from homemade cheese using in vitro assays, and these bacteria demonstrated susceptibility against antibiotics, enzymatic activity, and L‐lactate formation. The safety assessment results revealed that there is no evidence of toxicity caused by *L. lactis* IDCC 2301. *L. lactis* strains are considered food‐grade organisms, and they are used in a variety of food and feed preparations.

For further characterization, the *L. lactis* strain was grown at 37°C for 48 h and the carbohydrate consumption patterns were monitored. Forty‐nine selected carbohydrate sources were used, but after incubation, the strain fermented 14 carbohydrates, namely glucose, galactose, fructose, ribose, mannose, N‐acetyl‐glucosamine, esculin, cellobiose, maltose, sucrose, trehalose, gentiobiose, amygdaline, salicin, and amidon; moreover, amygdaline, salicin, and amidon were utilized very weakly. Armas *et al*. reported the same fermentation pattern of *L. lactis* LMG 7930 (Armas et al., 2017). Plumed‐Ferrer *et al*. also studied the same strain and reported the utilization of 8 carbohydrates out of 14 (Plumed‐Ferrer et al., 2013). They found that *L. lactis* could ferment ribose, arbutin, salicin, cellobiose, maltose, trehalose, saccharose, and gentiobiose. For the actual virulence factors, this difference might not be considered, although high carbohydrate fermentation activity may be a proof of better survival of the organisms.

From a genomic perspective, analysis of the complete genome of lactic acid‐producing bacteria is of great interest to scientists to know the pathogenicity and usefulness. To separate antibiotic susceptible strains from antibiotic‐resistant organisms, EFSA has clear guidelines for measuring the microbial cutoff values (Połka et al., 2016). The MIC distribution of the organisms can be used to determine the microbial cutoff values (Kim et al., 2018). Hence, in our study, we analyzed the genomic sequence, and no pathogenic gene or similar gene, which has antibiotic resistance, was identified through the MIC test. The cutoff values, which indicate the upper limit of the MIC distribution of the wild‐type *L. lactis,* were also reasonable with the nine selected antibiotics, which are comparable with different species of *Lactobacillus*. Moreover, all the tested antibiotics showed MICs ranging from 0.125 to 16 µg/mL, indicating the susceptibility of *L. lactis* IDCC 2301 to the antibiotics tested. Similarly, Kim et al., studied the susceptibility of *Bifidobacterium* spp. and found that the selected strain was susceptible to the applied antibiotic and the MIC range was from 0.01 to 4 µg/mL (Kim et al., 2018).

Bacterial hemolysin is responsible for the hemolytic activity, and it induces red blood cell lysis and consequently mild to severe infection by various pathogens (Nodzo et al., 2014). Therefore, before selecting probiotics, hemolytic activity analysis is very crucial (Sorokulova et al., 2008). In this regard, the 2002 FAO/WHO guidelines on probiotics safety considerations state that “if the strain under evaluation belongs to a species with known hemolytic potential, determination of hemolytic activity is required (FAO/WHO, 2002).” Although *Lactobacillus* spp. are used frequently in dairy industries, some strains can potentially behave as pathogenic microorganisms similar to common commensal microorganisms. Observation of physical changes by hemolytic activity after culturing the selected microorganism on an animal or human blood‐containing medium is commonly used to evaluate the hemolytic activity. In this study, *L. lactis* IDCC 2301 showed no hemolytic activity on sheep blood agar plates.

Biogenic amines, decarboxylation of amino acid products, and low molecular weight compounds are produced by lactic acid bacterial fermentation (Barbieri et al., 2019). These compounds have some negative health effects when the amine‐metabolizing power of the human body deteriorates (Alvarez & Moreno‐Arribas, 2014). These amines can be formed in fermented food during fermentation or decarboxylase activity by bacteria. However, the type and level of biogenic amines formed in food products depend on the composition of the food and the growth of bacteria during the processing and storage of foods. Protein‐rich fermented foods contain a high amount of biogenic amines (Jeong & Lee, 2015). For food safety purposes, tyramine and histamine are considered the most important amino acids and are responsible for various poisoning in fish and food‐induced migraines (Ban et al., 2020). In our study, biogenic amines, including tyramine, histamine, putrescine, 2‐phenethylamine, and cadaverine, were not detected in *L. lactis* IDCC 2301.

From the enzymatic activity perspective, the screened *L. lactis* strain was determined using the semi‐quantitative API ZYM system. β‐Glucosidase of lactic acid bacteria can hydrolyze glucose conjugates from plants, forming a variety of secondary metabolites. These metabolites function as health‐promoting substances (e.g., antioxidants) (Michlmayr et al., 2013). However, the enzymatic activity of β‐glucuronidase is important; it can indicate whether the carcinogenic compounds are present or not. It has been reported that β‐glucuronidase produced by microorganisms can develop toxic steroids, such as estrogen, or carcinogenic compounds, resulting in an increased risk for colorectal cancer (Kim & Jin, 2001). In this study, we confirmed *L. lactis* IDCC 2301 has no β‐glucuronidase activity. The leucine arylamidase activity of the selected strain of *L. lactis* was similar to that reported by Requena *et al*. and Otero *et al*. (Otero et al., 2001; Requena et al., 1991). However, the activities of valine and cystine arylamidase were not detected. These results are consistent with those of several studies (Otero et al., 2001; Requena et al., 1991; Tzanetakis & Litopoulou‐Tzanetaki, 1989). Requena *et al*. and Otero *et al*. reported similar results and found that the activity of leucine arylamidase was high in some strains of *L. plantarum* and *L. casei subsp. casei* (Otero et al., 2001; Requena et al., 1991). Considering all these results, *L. lactis* IDCC 2301 could be suitable probiotics for industrial application with respect to safety issues.

## 5 CONCLUSION

The utilization of probiotics in the food industry is gaining popularity, especially in the dairy sector. However, safety evaluation is the most important criterion before using the selected probiotic for human consumption. For safety assessment, antibiotic susceptibility, biogenic amine formation, and hemolytic activities need to be considered according to the 2002 FAO/WHO guideline. In this study, we evaluated the safety parameters of *L. lactis* IDCC 2301 isolated from homemade cheese. However, we did not find any antibiotic‐resistant gene through antibiotic susceptibility testing. Moreover, no biogenic compounds were produced after 24 h of incubation with the formation of only L‐lactate (18.27 ± 0.40 mg/mL). Poor hemolytic activity is considered a low risk for humans, and fortunately, in our study, the strain showed negative activity on sheep blood agar. Depending on the antibiotic, MIC values ranged from 1 (erythromycin and clindamycin) to 64 (kanamycin). *L. lactis* 2301 showed some enzymatic activities, but, importantly, β‐glucuronidase activity was absent. Hence, *L. lactis* IDCC 2301 can be used safely as a starter organism in cheese and other fermentation processes because they did not produce biogenic amines and did not show any hemolytic activity.

## CONFLICTS OF INTEREST

All authors declare that they have no competing interests.

## ETHICS APPROVAL

Not applicable.

## CONSENT FOR PUBLICATION

All authors read and approved the final manuscript for publication.

## Data Availability

Data are openly available in a public repository that issues datasets with DOIs.
